# Association of advanced lung cancer inflammation index with all-cause and cardiovascular mortality in US patients with asthma

**DOI:** 10.3389/fnut.2025.1525115

**Published:** 2025-06-27

**Authors:** Jiatao Li, Huanhuan Liu, Bin Xu

**Affiliations:** ^1^Department of Emergency Medicine, Beijing Tiantan Hospital, Capital Medical University, Beijing, China; ^2^Department of Surgery, International Medical Service, Peking Union Medical College Hospital, Beijing, China

**Keywords:** asthma, inflammation, advanced lung cancer inflammation index, NHANES, mortality

## Abstract

**Background:**

Asthma poses a significant global health challenge, representing a chronic respiratory disorder marked by airway inflammation. The advanced lung cancer inflammation index (ALI) served as a comprehensive index to assess inflammation. However, few studies have investigated the association between ALI and both all-cause and cardiovascular mortality in US patients with asthma.

**Methods:**

We used data from the National Health and Nutrition Examination Survey (NHANES) to explore the association of ALI with all-cause and cardiovascular mortality in US patients with asthma. This study used Kaplan–Meier curves to examine the ALI index's impact on asthma patients' survival. We applied weighted Cox models and restricted cubic splines (RCS) analysis to assess the ALI-mortality link, identifying non-linear thresholds with a recursive algorithm. Subgroup analyses and sensitivity analyses were conducted, excluding those with missing covariates and cancer patients.

**Results:**

A total of 6,211 asthma patients were enrolled and categorized into three groups based on ALI tertiles. The risk of all-cause mortality decreased as ALI increased in the fully adjusted multivariate Cox regression analysis; the hazard ratio (HR) is 0.95 (95% CI: 0.91–0.99, *P* = 0.01). Compared with the lowest ALI group, T1, the fully adjusted HR values for ALI and all-cause mortality in T2, T3 were 0.68 (95% CI: 0.55–0.85, *P* < 0.001), 0.53 (95% CI: 0.41–0.68, *P* < 0.001). The risk of cardiovascular mortality was also lower in the groups of T2 (HR: 0.84, 95% CI: 0.55–1.28) and T3 (HR: 0.47, 95% CI: 0.31–0.71, *P* for trend < 0.001), respectively. In addition, the results of the subgroup analyses were robust.

**Conclusions:**

This cohort study demonstrated the higher accuracy of ALI in predicting mortality in asthma patients, highlighting its important clinical value of ALI in risk assessment and prognosis evaluation.

## Introduction

Asthma is one of the most common chronic non-communicable diseases worldwide, affecting around 300 million individuals globally (1). Recent studies estimate that about 10% of children and teens have asthma symptoms worldwide, while the rate in adults is 6%−7% (2–4). Despite considerable advancements in asthma management, the disease continues to be a significant contributor to global mortality and disability, significantly impacting both all-cause and cardiovascular disease (CVD)-related deaths (5, 6). Consequently, asthma is a major global health issue, with researchers actively seeking to improve outcomes by addressing its risk factors, aiming for better disease management and patient prognosis.

Asthma is a chronic respiratory condition characterized by airway inflammation and hyperreactivity (7). The etiology and pathological progression of asthma were complex, with studies indicating that inflammation was involved in the occurrence and development of asthma (8–10). Higher inflammation levels are linked to the severity of asthma and imply a higher risk of mortality (11). In the early stages of asthma exacerbations, neutrophils play a crucial role by releasing various proinflammatory mediators, thereby contributing to airway inflammation (12). Sustained inflammation, driven by factors such as TNF and CRP, can lead to insulin resistance, which may cause weight loss and lower albumin levels (13, 14). Factors such as malnutrition and inflammation are all associated with hypoalbuminemia and adverse outcomes in various diseases (15). Furthermore, some research supported the notion that dietary practices and nutritional status can impact the incidence and prognosis of asthma (16, 17). Thus, relying on inflammation markers to predict asthma outcomes may miss important details. New indicators are clearly needed to provide a more complete picture of asthma patients' prognoses.

The advanced lung cancer inflammation index (ALI), which integrates body weight, albumin levels, and the neutrophil-to-lymphocyte ratio (NLR), was initially designed to gauge systemic inflammation in cancer patients (18–20). Additionally, the use of the ALI has been broadened to encompass several chronic conditions linked to nutritional imbalances and inflammatory processes (21–24). This extension highlights the versatility of the ALI in assessing inflammation across a range of health issues beyond cancer, potentially offering a valuable tool for monitoring and managing these chronic diseases. Since asthma is thought to be associated with inflammation (8), our study employed ALI to assess inflammation levels in asthma individuals and to explore its relationship with all-cause and cardiovascular disease (CVD) mortality in this group.

The primary objective of this research was to explore the possible connection between ALI and mortality rates, including all-cause and cardiovascular deaths, in individuals who have asthma. This investigation aims to provide a fresh viewpoint and significant understanding regarding the prognosis and risk assessment of individuals with asthma.

## Materials and methods

### Study population

**Figure 1:**
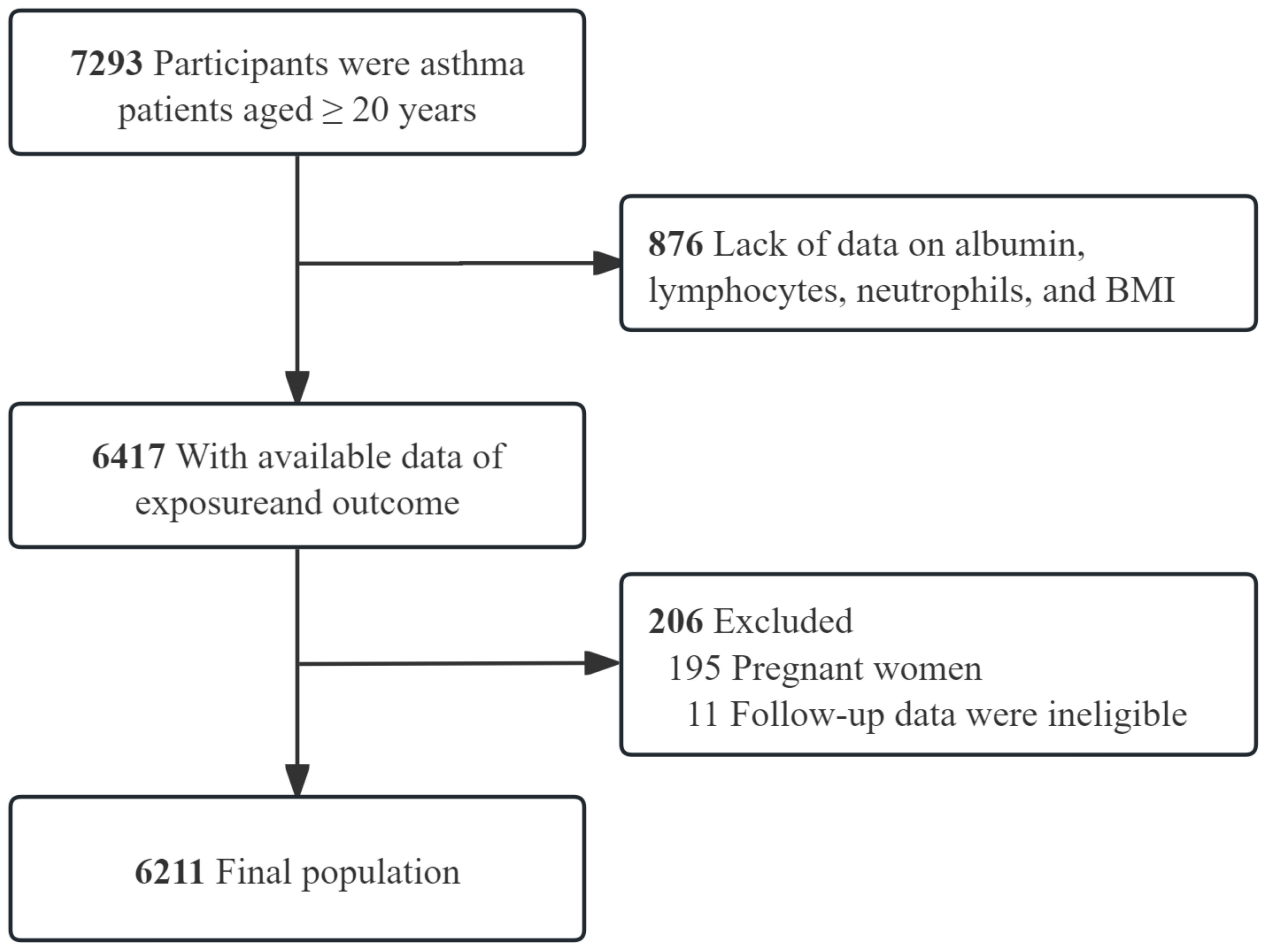
Inclusion and exclusion process for the final analysis was based on the 1999–2018 National Health and Nutrition.

NHANES, a cross-sectional survey by the National Center for Health Statistics (NCHS), employs a stratified multistage random sampling design (25, 26). The survey comprises a household interview and a medical examination at a mobile examination center (MEC). This retrospective analysis utilized NHANES data from 1999 to 2018, focusing on 7,293 asthma patients aged ≥20 years, 876 individuals were excluded due to incomplete data on albumin, lymphocytes, neutrophils, or BMI, leaving 6,417 participants with complete exposure and outcome data. Subsequent exclusions comprised 206 individuals, including 195 pregnant women and 11 with ineligible follow-up data, resulting in a final study population of 6,211 participants (Figure 1).

### Determination of ALI

ALI consists of albumin, BMI, and NLR, defined as albumin^*^BMI/NLR (neutrophil counts divided by lymphocyte counts). The Beckman Coulter MAXM instrument measured blood cell counts, and the DCX800 method measured albumin concentration. Based on the tertiles of ALI, patients with asthma were categorized into three groups: T1, T2, and T3.

### Mortality

Mortality data were obtained by linking the cohort database to the National Death Index as of December 31, 2019. All-cause mortality was determined to be death from any cause. Based on the International Statistical Classification of Diseases 10th Edition, CVD mortality includes the following disease codes: I00-I09 (acute rheumatic fever and chronic rheumatic heart diseases), I11 (hypertensive heart disease), I13 (hypertensive heart and renal disease), I20-I25 (ischemic heart diseases), I26-I28 (pulmonary embolism and other acute pulmonary heart diseases), I29 (various cardiovascular diseases caused by different reasons), I30-I51 (other forms of heart disease), and I60-I69 (cerebrovascular diseases).

### Definition of asthma

According to the NHANES questionnaire, patients who were told by a doctor that they had asthma and answered “yes” to the question, “Have you been told by a doctor that you have asthma?” were considered asthmatic (27, 28).

### Assessment of covariables

The study controlled for potential confounders including age, sex, race, education level, and family income to poverty ratio (PIR; < 1.3, 1.3–3.5, ≥3.5), BMI (<25, 25–30, ≥30), smoking status, diabetes, hypertension, and cardiovascular disease (CVD). Race was categorized as non-Hispanic White, Black, Mexican American, Other Hispanic, and Other. Education levels were classified into fewer than 9 years, 9–12 years, and more than 12 years. Smoking status was classified as never, former, and current smokers. Diabetes was diagnosed based on a medical diagnosis, HbA1c >6.5%, fasting glucose ≥7.0 mmol/L, random/2-h oral glucose tolerance test ≥11.1 mmol/L, or the use of diabetes medication/insulin. Hypertension was defined by self-report, average systolic blood pressure ≥140 mmHg, diastolic blood pressure ≥90 mmHg, or the use of antihypertensive medication (29). Physical activity (PA) was quantified as the weekly time spent on activities such as walking, biking, household chores, work-related tasks, and recreational pursuits; individuals who did not exercise were recorded as zero. A reported history of heart failure, coronary heart disease, angina, myocardial infarction, or stroke identified CVD. Inhaled corticosteroid use was extracted from prescription records.

### Statistical analyses

Our study incorporated the MEC weights in compliance with the NHANES analysis guidelines, which provide instructions on employing survey sample weights (30). We calculated sampling weights period-specifically. From 1999 to 2002, a factor of 2/10 was applied to the 4-year MEC weight; from 2003 to 2018, a factor of 1/10 was used for the 2-year MEC weight. The National Death Index, updated every 4 years, provided the latest follow-up data through December 31, 2019. Follow-up for each participant spanned from their MEC examination date to the date of death or the end of follow-up on December 31, 2019. Given minimal missing data (0%−8%) across variables, we employed a multivariable single imputation method using Bayesian Ridge regression for iterative imputation of missing values, aligning with the methodology recommended by van Buuren and Groothuis–Oudshoorn in 2011. This approach ensures statistically robust handling of missing data, complying with current field standards.

Statistical analyses followed NHANES guidelines, including sample weights and stratification. Continuous variables were expressed as mean ± SD for normally distributed data and median (IQR) for non-normally distributed data. Analysis of variance (ANOVA) assessed baseline differences in continuous variables, and chi-square tests assessed categorical variables expressed as counts (percentages).

Kaplan–Meier analyses explored the association between ALI and all-cause mortality and CVD mortality in patients with asthma. Multivariate Cox regression was used to analyze the effect of a 10-unit increase in ALI on mortality, thus accurately quantifying the prognostic impact of ALI on asthma. Multivariate Cox regression analyses adjusted for covariates explored the effect of ALI on mortality. Results were expressed as HR (95% CI). Three models were used: Model 1 (unadjusted), Model 2 (adjusted for demographic and lifestyle factors), and Model 3 (further adjusted for clinical conditions). The analyses used the weights recommended by NHANES.

We assessed the non-linear relationship between ALI and mortality using restricted cubic spline (RCS) analysis and multivariate cox models. A recursive algorithm identified the inflection point of the non-linear relationship, and a segmented cox model assessed the threshold effect. To enhance the robustness of the study, we performed stratified analyses of potential interactions between ALI and variables such as age, sex, BMI, diabetes, and inhaled corticosteroid use. To validate the robustness of our findings, we conducted a comprehensive sensitivity analysis. Initially, we excluded participants with missing covariate data, followed by the removal of individuals with a cancer diagnosis. Subsequently, we employed a multivariate cox proportional hazards model and RCS analysis to explore the association between ALI and mortality outcomes. We also applied the E-value method by VanderWeele and Ding (31) to evaluate the robustness of our findings against unmeasured confounding. To evaluate the predictive performance of ALI and NLR for all-cause and CVD mortality in asthma patients, receiver operating characteristic (ROC) curve analyses were conducted. All analyses were performed using R Statistical Software (Version 4.2.2, https://www.R-project.org, The R Foundation) and Free Statistics analysis platform (Version 2.0, Beijing, China).

## Results

### Baseline characteristics

In our study of asthma patients, the cohort included 6,211 participants with a weighted total of 27,739,218 individuals. According to the tertiles of ALI, the patients were divided into three groups: T1 group (*n* = 2,070), T2 group (*n* = 2,070), and T3 group (*n* = 2,071). The overall mean age was 45.32 years, and there was a notable difference in age across groups (*P* < 0.001). The majority of participants were female (58.43%) and Non-Hispanic White (70.63%), with significant racial distribution differences across three groups (*P* < 0.001). Family income, education level, smoking status, physical activity, and BMI also showed significant variations (*P* < 0.05 for all; Table 1). The essential characteristics of excluded and included participants are presented in Supplementary Table S1.

**Table 1:** The demographic characteristics of participants (Weighted).

**Characteristics**	**Overall**	**T1 (2.89, 52.01)**	**T2 (52.01, 77.24)**	**T3 (52.01, 2,186.00)**	***P***
	***n*** = **6,211**	***n*** = **2,070**	***n*** = **2,070**	***n*** = **2,071**	
*n*	27,739,218	9,545,823	9,942,045	8,251,349	
ALI [median (IQR)]	61.96 (45.81, 82.48)	40.07 (31.24, 46.34)	63.36 (57.76, 69.83)	98.32 (86.15, 119.52)	<0.001
Age (year) (mean, SD)	45.32 (16.72)	47.94 (17.85)	44.32 (15.88)	43.51 (15.96)	<0.001
**Sex**, ***n*** **(%)**					
Male	2,642 (41.57)	944 (41.66)	858 (41.67)	840 (41.33)	0.981
Female	3,569 (58.43)	1,126 (58.34)	1,212 (58.33)	1,231 (58.67)	
**Race**, ***n*** **(%)**					
Non-Hispanic White	3,001 (70.63)	1,172 (76.51)	1,076 (73.93)	753 (59.84)	<0.001
Non-Hispanic Black	1,460 (11.95)	308 (6.78)	396 (9.44)	756 (20.97)	
Mexican American	638 (4.82)	201 (4.35)	226 (4.81)	211 (5.36)	
Other Hispanic	570 (5.94)	191 (5.60)	204 (6.01)	175 (6.24)	
Other race	542 (6.66)	198 (6.75)	168 (5.81)	176 (7.59)	
**Poverty income ratio**, ***n*** **(%)**					
≤1.30	2,228 (25.83)	752 (25.90)	700 (23.09)	776 (29.06)	0.001
1.31–3.50	2,208 (34.71)	722 (33.52)	723 (34.42)	763 (36.42)	
>3.50	1,775 (39.46)	596 (40.58)	647 (42.49)	532 (34.52)	
**Education level**, ***n*** **(%)**					
Less than high school	1,437 (15.66)	501 (16.10)	441 (13.49)	495 (17.75)	0.001
High school or equivalent	1,392 (22.67)	491 (24.50)	431 (20.61)	470 (23.06)	
Above high school	3,382 (61.67)	1,078 (59.40)	1,198 (65.90)	1,106 (59.19)	
**Smoke status**, ***n*** **(%)**					
Never smoker	3,035 (49.37)	913 (45.53)	1,041 (51.11)	1,081 (51.71)	0.012
Former smoker	1,646 (26.22)	609 (27.50)	519 (25.52)	518 (25.59)	
Current smoker	1,530 (24.41)	548 (26.97)	510 (23.37)	472 (22.70)	
Physical activity (mean, SD)	676.72 (1,322.10)	644.84 (1,277.90)	642.52 (1,177.93)	754.81 (1,519.33)	0.041
BMI (mean, SD)	29.95 (7.83)	26.80 (6.05)	29.92 (7.33)	33.63 (8.62)	<0.001
**CVD**, ***n*** **(%)**					
No	5,226 (87.52)	1,681 (85.41)	1,781 (89.64)	1,764 (87.40)	0.003
Yes	985 (12.48)	389 (14.59)	289 (10.36)	307 (12.60)	
**Diabetes**, ***n*** **(%)**					
No	4,977 (85.70)	1,668 (86.99)	1,670 (85.98)	1,639 (83.87)	0.028
Yes	1,234 (14.30)	402 (13.01)	400 (14.02)	432 (16.13)	
**Hypertension**, ***n*** **(%)**					
No	3,339 (60.02)	1,086 (59.68)	1,200 (63.26)	1,053 (56.49)	0.002
Yes	2,872 (39.98)	984 (40.32)	870 (36.74)	1,018 (43.51)	
**Inhaled corticosteroid**, ***n*** **(%)**					
No	5,418 (87.75)	1,770 (86.83)	1,815 (87.63)	1,833 (88.95)	0.30
Yes	793 (12.25)	300 (13.17)	255 (12.37)	238 (11.05)	

For statistical analysis, continuous variables were presented as mean ± standard deviation or median (interquartile range), and categorical variables were expressed as percentages. All measures were appropriately weighted.

SD, standard deviation; ALI, advanced lung cancer inflammation index; BMI, body mass index; CVD, cardiovascular disease.

### Kaplan–Meier analysis

**Figure 2:**
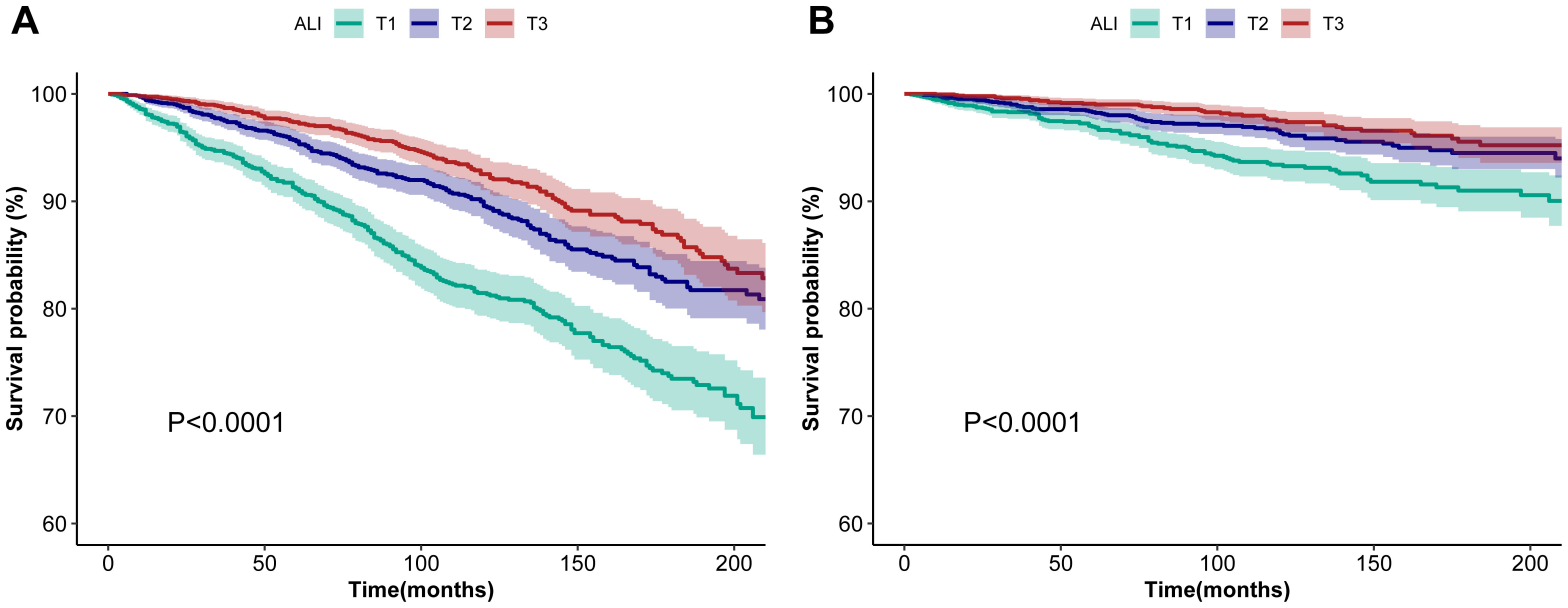
Kaplan–Meier survival curves of ALI impact on long-term all-cause **(A)** and CVD **(B)** mortality in patients with asthma. **(A)** and **(B)** show Kaplan–Meier survival curves for ALI patients stratified by severity (T1, T2, and T3), depicting associations with all-cause **(A)** and CVD-specific mortality **(B)**. Shaded areas indicate 95% confidence intervals, and log-rank tests (*P* < 0.0001) confirm significant survival differences across ALI stages, underscoring its prognostic value. ALI, advanced lung cancer inflammation index; CVD, cardiovascular disease; T1, tertiles 1; T2, tertiles 2; T3, tertiles 3.

Among the 6,211 participants, a total of 865 all-cause deaths and 259 CVD deaths were documented. Kaplan–Meier analysis was utilized to preliminary evaluate the association between ALI and all-cause and CVD mortality in asthma patients. Figure 2 illustrates that higher ALI is associated with decreased all-cause and CVD mortality in asthma patients (*P* < 0.001).

### Multivariate cox analysis

Table 2 from our study presents the outcomes of three Cox proportional hazards models, with Model 3 being the most adjusted and, thus providing the most reliable estimates by accounting for a broader range of potential confounders. Model 3 results underscore the critical finding of our study: a higher ALI is inversely associated with mortality risk in asthmatic patients from the NHANES 1999–2018 cohort. Model 3 indicated that for each 10-unit increase in ALI, there was a significant decrease in the risk of all-cause mortality, with a hazard ratio (HR) of 0.95 (95% CI: 0.91–0.99; *P* = 0.01). When compared to the lowest ALI group (T1), the middle (T2), and highest (T3) groups exhibited progressively lower risks of all-cause mortality, with HRs of 0.68 (95% CI: 0.55–0.85; *P* < 0.001) and 0.53 (95% CI: 0.41–0.68; *P* < 0.001), respectively (*P* for trend < 0.001). A similar trend was observed for CVD mortality. Model 3 indicated that for each 10-unit increase in ALI, there was a significant decrease in the risk of CVD mortality, with a hazard ratio (HR) of 0.90 (95% CI: 0.85–0.96; *P* < 0.001). When compared to the lowest ALI group (T1), the middle (T2), and highest (T3) groups exhibited progressively lower risks of CVD mortality, with HRs of 0.84 (95% CI: 0.55–1.28; *P* = 0.428) and 0.47 (95% CI: 0.31–0.71; *P* < 0.001), respectively (*P* for trend < 0.001).

**Table 2:** Association of ALI with all-cause and CVD mortality in asthma patients in the NHANES 1999–2018 cohort.

**Variable**	**No. death/total**	**Model 1**		**Model 2**		**Model 3**	
		**HR (95% CI)**	***P*** **-value**	**HR (95% CI)**	***P*** **-value**	**HR (95% CI)**	***P*** **-value**
**All-cause mortality**							
ALI, Per 10 U increment	865/6,211	0.91 (0.87, 0.95)	<0.001	0.95 (0.92, 0.99)	0.014	0.95 (0.91, 0.99)	0.01
ALI tertiles							
T1	435/2,070	1 (Ref)		1 (Ref)		1 (Ref)	
T2	245/2,070	0.52 (0.43, 0.64)	<0.001	0.72 (0.58, 0.90)	0.003	0.68 (0.55, 0.85)	<0.001
T3	185/2,071	0.44 (0.36, 0.54)	<0.001	0.59 (0.47, 0.74)	<0.001	0.53 (0.41, 0.68)	<0.001
*P* for trend			<0.001		<0.001		<0.001
**CVD mortality**							
ALI, Per 10 U increment	259/6,211	0.89 (0.83, 0.94)	<0.001	0.94 (0.89, 0.98)	0.006	0.90 (0.85, 0.96)	<0.001
ALI tertiles							
T1	127/2,070	1 (Ref)		1 (Ref)		1 (Ref)	
T2	78/2,070	0.7 (0.47, 1.05)	0.086	1 (0.67, 1.50)	0.992	0.84 (0.55, 1.28)	0.428
T3	54/2,070	0.45 (0.31, 0.65)	<0.001	0.61 (0.42, 0.88)	0.009	0.47 (0.31, 0.71)	<0.001
*P* for trend			<0.001		0.021		<0.001

ALI, advanced lung cancer inflammation index; Ref, reference; HR, hazard ratios; CI, confidence interval; CVD, cardiovascular disease; PIR, poverty income ratio; BMI, body mass index.

Model 1: unadjusted.

Model 2: age, sex, race, PIR, education level, physical activity.

Model 3: model 2 + smoking status, BMI, CVD, diabetes, hypertension, inhaled corticosteroid.

### Non-linear relationships

**Figure 3:**
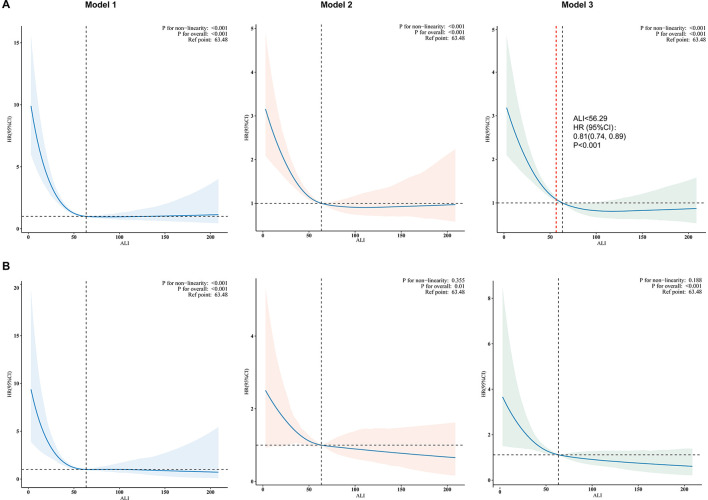
The association between the ALI and all-cause **(A)** and CVD **(B)** mortality in patients with asthma. Model 1 was unadjusted; Model 2 adjusted for age, sex, race, PIR, education level, and physical activity; Model 3 further adjusted for smoking status, BMI, CVD, diabetes, hypertension, and inhaled corticosteroid use. Notably, 99% of the data are displayed in the figure. The solid line depicts the estimated values, accompanied by shaded ribbons representing the 95% confidence intervals. The horizontal dotted line indicates an odds ratio of 1.0, serving as the reference line. Furthermore, the red vertical dotted line signifies the ALI threshold value of 56.29, while the black vertical dotted line marks the reference point, set at an ALI value of 63.48. ALI, advanced lung cancer inflammation index; CVD, cardiovascular disease; PIR, poverty income ratio; BMI, body mass index.

Our study used weighted RCS analysis to explore the relationship between ALI and mortality in asthmatic patients (Figure 3). Figure 3A depicts the dose-response association between ALI and all-cause mortality across three progressively adjusted models. All models revealed significant non-linearity (*P* for non-linearity <0.001), with a consistent L-shaped pattern. In contrast, Figure 3B demonstrates a linear association between ALI and CVD mortality (*P* for non-linearity >0.05). For all-cause mortality, a pivotal threshold was identified at ~56.29 units of ALI. Below this threshold, a significant inverse association was observed between ALI and all-cause mortality (HR = 0.81, 95% CI: 0.74–0.89, *P* < 0.001 in Model 3). Conversely, no significant association was detected for ALI values ≥56.29 (HR = 1.00, 95% CI: 0.98–1.03, *P* = 0.9 in Model 3). These results remained consistent across all three models, underscoring the robustness of the observed relationship (Table 3).

**Table 3:** Association of ALI with all-cause mortality in patients with asthma using two-piecewise regression models.

**The inflection point of ALI**	**All-cause mortality**					
	**ALI, Per 10U increment**					
	**Model 1**		**Model 2**		**Model 3**	
	**HR (95% CI)**	***P*** **-value**	**HR (95% CI)**	***P*** **-value**	**HR (95% CI)**	***P-*** **value**
<56.29	0.65 (0.58, 0.72)	<0.001	0.81 (0.74, 0.89)	<0.001	0.81 (0.74, 0.89)	<0.001
≥56.29	1.01 (0.99, 1.03)	0.22	1.01 (0.99, 1.02)	0.51	1.00 (0.98, 1.03)	0.9

ALI, advanced lung cancer inflammation index; HR, hazard ratios; CI, confidence interval; CVD, cardiovascular disease; PIR, poverty income ratio; BMI, body mass index.

Model 1: unadjusted.

Model 2: age, sex, race, PIR, education level, physical activity.

Model 3: model 2 + smoking status, BMI, CVD, diabetes, hypertension, inhaled corticosteroid.

### Stratified analyses

**Figure 4:**
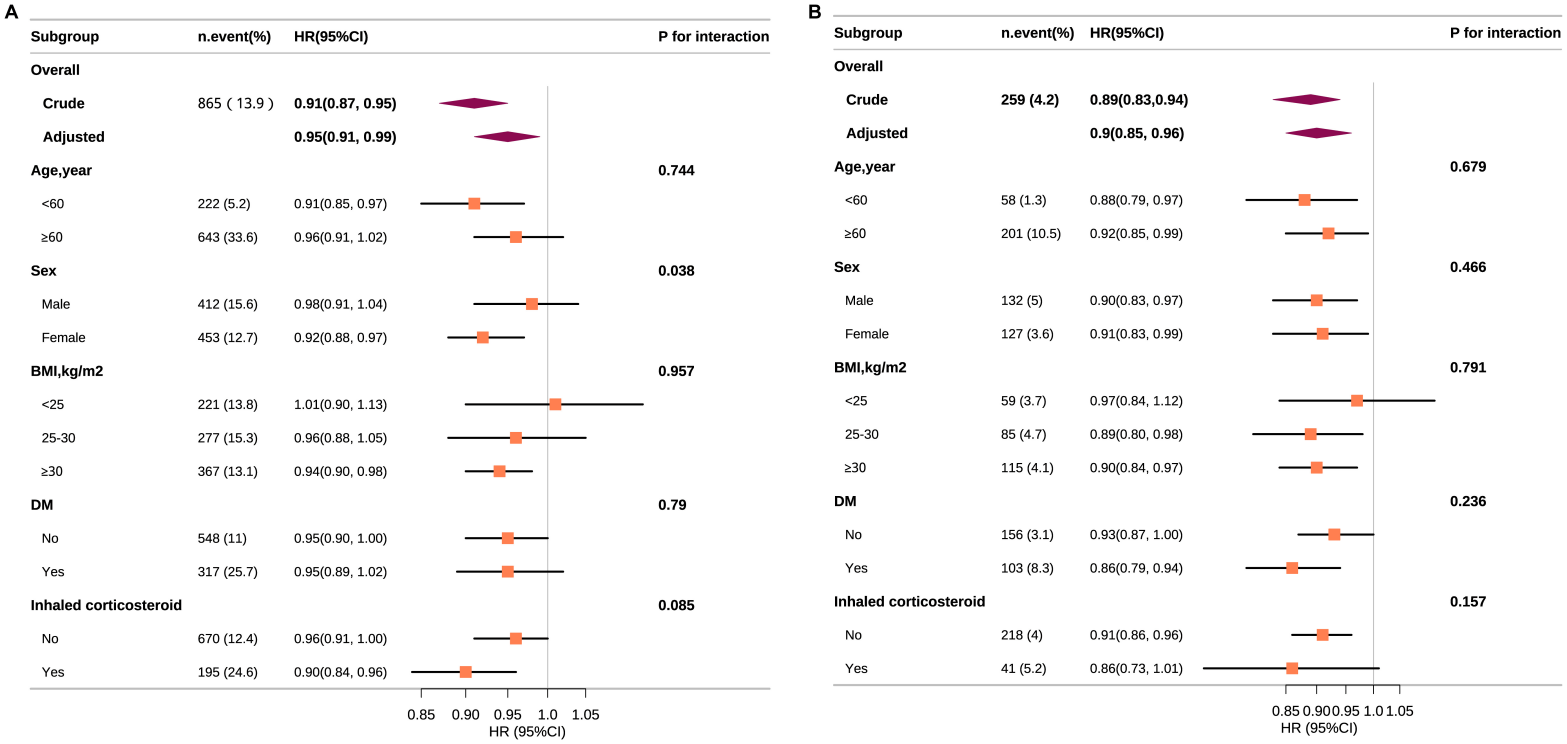
Association between ALI and all-cause **(A)** and CVD **(B)** mortality according to general characteristics. Except for the stratification component itself, each stratification factor was adjusted for all other variables (age, sex, race, PIR, education level, physical activity, smoking status, BMI, CVD, diabetes, hypertension, and inhaled corticosteroid). Squares represent the HRs and horizontal lines represent 95% CIs. Diamonds represent the overall HR, and the outer points of the diamonds represent the 95% CI. CVD, cardiovascular disease; PIR, poverty income ratio; BMI, body mass index; CI, confidence interval; HR, hazard ratio.

Figure 4 delineates the stratified associations of ALI with all-cause (Panel A) and CVD mortality (Panel B) across various subgroups, accounting for numerous confounders. In all-cause mortality, ALI showed significant protective effects among females (HR: 0.92, 95% CI: 0.88–0.97), those with BMI ≥30 kg/m^2^ (HR: 0.94, 95% CI: 0.90–0.98), and users of inhaled corticosteroids (HR: 0.90, 95% CI: 0.84–0.96). For CVD mortality, significant risk reductions were noted in individuals under 60 years (HR: 0.88, 95% CI: 0.79–0.97), males (HR: 0.90, 95% CI: 0.83–0.97), and diabetics (HR: 0.86, 95% CI: 0.79–0.94). No significant interactions were found in subgroups after stratification according to age, BMI, and diabetes. Although the interaction for sex in all-cause mortality was statistically significant (*P* < 0.05), it may not be clinically meaningful given multiple testing and the consistent direction of the associations.

### Sensitivity analysis

Elevated ALI levels were consistently inversely associated with all-cause and CVD mortality across sensitivity analyses. Excluding participants with missing data or cancer, each 10-unit ALI increment reduced all-cause mortality by 6%−7% and CVD mortality by 10%−11%. The highest ALI tertiles (T3) showed the strongest protective effects, with significant reductions in mortality compared to the lowest tertiles (T1). Trend tests confirmed dose-response relationships, highlighting the robustness of these findings (Supplementary Tables S2, S3). Supplementary Figure S1, we performed weighted restricted cubic spline (RCS) regression analyses, excluding participants with missing covariate data or a history of cancer. The fully adjusted model revealed a significant non-linear association (*P* for non-linearity <0.001), characterized by a consistent L-shaped curve for all-cause mortality. Threshold values for ALI were identified at 55.44 and 54.19, respectively. In contrast, the relationship between ALI and CVD mortality exhibited a linear trend (*P* for non-linearity >0.05). *E*-value analyses demonstrate that the observed inverse associations between ALI and mortality are robust to potential unmeasured confounding, particularly for the highest ALI tertile (*E*-values > 3), suggesting a dose-dependent protective effect unlikely to be fully explained by confounding factors (Supplementary Table S4).

The ROC curve analysis revealed that ALI demonstrated a moderate predictive ability for all-cause mortality, with an AUC of 0.630 (95% CI: 0.608–0.651; *P* < 0.05). For CVD mortality, the AUC for ALI was 0.619 (95% CI: 0.582–0.656), while NLR yielded a comparable AUC of 0.621 (95% CI: 0.584–0.658). The difference between ALI and NLR in predicting CVD mortality was not statistically significant (*P* > 0.05). These findings suggest that ALI and NLR exhibit similar discriminative performance for CVD mortality, while ALI shows a statistically significant predictive value for all-cause mortality (Supplementary Figure S2).

## Discussion

This cohort study in the United States utilized ALI to evaluate the inflammatory status of asthma patients. The Cox model adjusted for multiple factors demonstrated that a rise in ALI correlated with decreased risk of all-cause mortality and CVD mortality for asthma patients. Furthermore, RCS analyses revealed a non-linear L-shaped relationship between ALI and all-cause mortality (inflection point: 56.29), whereas a linear association was observed between ALI and CVD mortality. This association remained consistent across age groups, BMI categories, DM, and inhaled corticosteroid use. These results suggest that lower levels of inflammation were associated with a lower risk of all-cause death and CVD death in individuals with asthma.

Asthma, characterized by chronic immune inflammation, is recognized for its impact on reducing life expectancy and is associated with an increasing mortality gap compared to the general population (32). Low-grade chronic inflammation may result in atherosclerosis, hypertension, diabetes, and obesity, which are all recognized as risk factors for cardiovascular disease and CVD mortality (11, 13). Asthma development is significantly influenced by inflammation, which is central to the condition (9). In asthma's progression, localized and systemic inflammatory responses are implicated (16, 33). In asthma, monocytes mature into macrophages, playing a vital role in the chronic inflammation characteristic of the disease (34). In the study by Ke et al. (35) it was noted that the inflammatory index NLR is positively associated with the prevalence of asthma; it also significantly correlated with all-cause mortality among asthma patients. The role of NLR indicators in respiratory diseases has aroused intense interest among researchers. Citu et al. (36) has pinpointed elevated NLR as independent predictor of unfavorable outcomes in COVID-19 patients.

Moreover, Fois et al. (37) have also reported higher NLR values in individuals with severe COVID-19. These findings indicate that inflammation adversely affects the prognosis of asthma patients, which is consistent with the results of our research. It is crucial to note that previous studies typically concentrate on single inflammatory markers, potentially failing to provide a complete picture of a patient's inflammatory profile. Beyond inflammation, nutrition is another area of focus. A growing body of evidence suggests a possible link between dietary factors and the development and progression of asthma (17, 38, 39). According to a literature review by Althoff et al. (40), asthma is associated with comorbid obesity, and the interplay among these diseases is not fully understood. Nygaard et al. (16) found inverse associations between the Dietary Approaches to Stop Hypertension scores and reduced general proinflammatory biomarkers, such as cytokines and growth factors, associated with asthma. Moreover, previous studies found a J-shaped or U-shaped relationship between BMI and mortality, with a BMI interval having the lowest mortality rate. Although the specific values may vary in different studies, a positive correlation existed between BMI and mortality after exceeding this threshold (41–43).

The ALI, a composite index that integrates measures of inflammation and nutrition, including BMI, serum albumin, and the neutrophil-to-lymphocyte ratio (NLR), was initially used to assess the mortality risk in lung cancer patients (44, 45). Studies have since applied the ALI to evaluate nutritional status and inflammation in various cancer types and chronic conditions (21, 46), underscoring its prognostic significance. In particular, Jafri et al. (18) demonstrated that ALI increases the survival odds against all-cause mortality in non-small cell carcinoma patients. A heart failure study indicated that higher ALI scores were linked to a lower risk of all-cause mortality and hospital readmission (23). Moreover, a study focusing on rheumatoid arthritis (RA) revealed that elevated ALI is significantly linked to a reduced risk of all-cause and cardiovascular mortality in RA patients (21).

Additionally, evidence indicates that ALI has an independent influence on both all-cause and cardiovascular mortality among hypertensive patients (22). It is significant that these studies consistently portray ALI as a beneficial factor, aligning with our findings. As far as we know, no previous study has investigated the relationship between ALI and mortality rates in asthma patients. Our research tentatively suggests that in asthma patients, an increase in ALI is associated with a reduced risk of all-cause and cardiovascular mortality.

The potential association between elevated ALI and decreased all-cause and cardiovascular mortality in patients with asthma can be analyzed from two aspects. On the one hand, inflammation's role is significant as it is a critical component of asthma and has been shown to contribute to its severity and outcomes (13, 14). Besides, BMI is a recognized risk factor for all-cause and CVD mortality (47–49); it is essential to factor it in when evaluating the connection between ALI and CVD mortality in patients with asthma (38). Our study used curve fitting to delineate the relationship between ALI and mortality, revealing an L-shaped association with a threshold saturation effect. We hypothesize that this may be due to increased BMI leading to more severe adverse outcomes, thereby diminishing the protective effect. Consequently, we controlled for BMI and used RCS to explore potential non-linear associations between ALI and mortality. However, it is essential to note that the relationship between ALI and mortality in asthma patients still requires further investigation, as no study to date has specifically addressed this association.

Our research has multiple strengths. Firstly, the large sample size enhanced our analysis's statistical power and broadened the generalizability of our results to a broader asthma patient population. Secondly, we utilized a range of statistical techniques to reduce the impact of confounding variables on our results, including multivariable cox regression, stratified analysis, and interaction analysis, ensuring our findings' robustness. Thirdly, ALI functioned as a comprehensive indicator, which was straightforward to obtain and calculate, making it highly practical for clinical practice. Lastly, we employed a fitted curve to quantify the relationship between ALI and all-cause and CVD mortality, identifying a critical inflection point.

Nevertheless, our study also has limitations. First, although inhaled corticosteroid use was rigorously adjusted as a covariate in all analyses, NHANES lacks granular data on treatment patterns, precluding stratified analyses to assess their potential modifying effects on the ALI-mortality association. Second, asthma severity and exacerbation history were unavailable in the database, which may introduce unmeasured confounding. Nevertheless, the various sensitivity and subgroup analyses performed confirmed the stability of the findings. Third, ALI was calculated using baseline measurements of albumin, BMI, and NLR. While this approach aligns with prior NHANES-based studies, the lack of serial biomarker data limits our ability to evaluate how dynamic changes in these components might influence ALI's prognostic value over time. Fourth, our cohort comprised US adults with self-reported asthma diagnoses, potentially underrepresenting global populations with diverse genetic, environmental, and healthcare backgrounds. Cross-ethnic and multi-regional validations are needed to confirm ALI's generalizability. Finally, given the observational nature of our study, establishing a definitive causal link between ALI and mortality remains unattainable. This investigation analyzed the link between ALI and mortality in adults from the US by utilizing publicly accessible databases. Future prospective investigations are warranted to further explore this topic.

## Conclusions

In conclusion, our study has tentatively established a substantial correlation between higher ALI and a reduced risk of all-cause and CVD mortality in patients with asthma. Our findings indicate that ALI exhibits an L-shaped, non-linear association with all-cause mortality, with critical inflection points at 56.29. Additionally, a linear association was observed between ALI and CVD mortality. These patterns suggest that ALI may have a complex relationship with mortality outcomes, which could have implications for its use as a prognostic tool in this patient population. However, these are early observations and would benefit from further investigation in larger, more diverse cohorts to validate the robustness of these relationships.

## Data Availability

The datasets presented in this study can be found in online repositories. The names of the repository/repositories and accession number(s) can be found at: https://www.cdc.gov/nchs/nhanes.htm.
