# Supplementary material for: Association of advanced lung cancer inflammation index with all-cause and cardiovascular mortality in US patients with asthma
Source: Front Nutr. 2025 Jun 27;12:1525115. doi: 10.3389/fnut.2025.1525115 (PMC12245702; doi:10.3389/fnut.2025.1525115)
Supplement: Supplementary file 1 [file Data_Sheet_1.pdf]

Supplementary Material

Supplementary Table S1. Essential characteristics of excluded and included participants.

Supplementary Table S2. Association of ALI with all-cause and CVD mortality in asthma patients in the NHANES 1999–2018 cohort, excluding individuals with missing covariate data.

Supplementary Table S3. Association of ALI with all-cause and CVD mortality in asthma patients in the NHANES 1999–2018 cohort, excluding individuals with cancer.

Supplementary Table S4. Sensitivity analysis using E-values.

Supplementary Figure S1. Association between ALI and all-cause and CVD mortality in asthma patients. (A, C) excluding individuals with missing covariate data. (B, D) excluding individuals with cancer.

Supplementary Figure S2. Comparative ROC analysis of ALI and NLR for predicting mortality outcomes.

Supplementary Table S1. Essential characteristics of excluded and included participants.

Characteristic	Total (n = 7293)	Excluded population (n = 1082)	Included population (n = 6211)
Age(year) (mean (SD))	47.7 ± 18.2	46.3 ± 20.3	48.0 ± 17.8
Sex, n(%)			
Male	2993 (41.0)	351 (32.4)	2642 (42.5)
Female	4300 (59.0)	731 (67.6)	3569 (57.5)
Race, n(%)			
Non-Hispanic White	3476 (47.7)	475 (43.9)	3001 (48.3)
Non-Hispanic Black	1789 (24.5)	329 (30.4)	1460 (23.5)
Mexican American	737 (10.1)	99 (9.1)	638 (10.3)
Other Hispanic	671 (9.2)	101 (9.3)	570 (9.2)
Other Race	620 (8.5)	78 (7.2)	542 (8.7)
Poverty income ratio , n(%)			
≤1.30	2424 (36.3)	366 (38.9)	2058 (35.9)
1.31-3.50	2349 (35.2)	340 (36.2)	2009 (35)
>3.50	1899 (28.5)	234 (24.9)	1665 (29)
Education level , n(%)			
Less than high school	1747 (24.0)	311 (29)	1436 (23.1)
High school or equivalent	1649 (22.7)	259 (24.1)	1390 (22.4)
Above high school	3883 (53.3)	504 (46.9)	3379 (54.5)
Smoke status, n(%)			
Never smoker	3596 (49.4)	564 (52.3)	3032 (48.9)
Former smoker	1890 (25.9)	244 (22.6)	1646 (26.5)
Current smoker	1799 (24.7)	271 (25.1)	1528 (24.6)
Physical activity (mean(SD))	651.5 ± 1332.0	502.8 ± 1199.8	677.4 ± 1352.1
BMI (mean (SD))	30.5 ± 8.1	31.0 ± 8.9	30.5 ± 8.0
CVD, n(%)			
No	6112 (83.8)	887 (82.1)	5225 (84.1)
Yes	1179 (16.2)	194 (17.9)	985 (15.9)
Diabetes, n(%)			
No	5644 (79.9)	667 (78.1)	4977 (80.1)
Yes	1421 (20.1)	187 (21.9)	1234 (19.9)
Hypertension, n(%)			
No	3953 (54.2)	614 (56.9)	3339 (53.8)
Yes	3337 (45.8)	465 (43.1)	2872 (46.2)
Inhaled corticosteroid, n(%)			
No	6397 (87.8)	982 (91.3)	5415 (87.2)
Yes	886 (12.2)	93 (8.7)	793 (12.8)

Supplementary Table S2. Association of ALI with all-cause and CVD mortality in asthma patients in the NHANES 1999–2018 cohort, excluding individuals with missing covariate data.

		All -cause mortality					
Variable	No.death/total	Model 1		Model 2		Model 3	
		HR (95%CI)	*P*-value	HR (95%CI)	*P*-value	HR (95%CI)	*P*-value
ALI, Per 10 U increment	782/5723	0.91(0.87, 0.96)	<0.001	0.95(0.91, 0.99)	0.027	0.94(0.90, 0.99)	0.014
ALI tertiles							
T1	388/1908	1(Ref)		1(Ref)		1(Ref)	
T2	224/1912	0.53(0.43, 0.65)	<0.001	0.72(0.58, 0.90)	0.004	0.68(0.54, 0.85)	<0.001
T3	170/1903	0.46(0.37, 0.56)	<0.001	0.59(0.47, 0.74)	<0.001	0.52(0.40, 0.67)	<0.001
P for trend			<0.001		<0.001		<0.001
		CVD mortality					
Variable	No.death/total	Model1		Model2		Model3	
		HR (95%CI)	*P*-value	HR (95%CI)	*P*-value	HR (95%CI)	*P*-value
ALI, Per 10 U increment	234/5723	0.89(0.84, 0.95)	<0.001	0.94(0.90, 0.99)	0.019	0.90(0.85, 0.96)	<0.001
ALI tertiles							
T1	112/1908	1(Ref)		1(Ref)		1(Ref)	
T2	72/1912	0.73(0.48, 1.12)	0.149	1.06(0.69, 1.62)	0.796	0.88(0.57, 1.36)	0.562
T3	50/1903	0.49(0.33, 0.71)	<0.001	0.66(0.45, 0.97)	0.035	0.49(0.32, 0.75)	0.001
P for trend			<0.001		0.071		0.002

Abbreviations: ALI, advanced lung cancer inflammation index; Ref, reference; HR, hazard ratios; CI, confidence interval; CVD, cardiovascular disease; PIR, poverty income ratio; BMI, body mass index.

Model1: unadjusted.

Model2: age, sex, race, PIR, education level, physical activity.

Model3: model2 + smoking status, BMI, CVD, diabetes, hypertension, inhaled corticosteroid.

Supplementary Table S3. Association of ALI with all-cause and CVD mortality in asthma patients in the NHANES 1999–2018 cohort, excluding individuals with cancer.

		All -cause mortality					
Variable	No.death/total	Model1		Model2		Model3	
		HR (95%CI)	*P*-value	HR (95%CI)	*P*-value	HR (95%CI)	*P*-value
ALI, Per 10 U increment	668/5539	0.91(0.87, 0.95)	<0.001	0.95(0.92, 0.98)	0.003	0.93(0.90, 0.97)	<0.001
ALI tertiles							
T1	318 /1784	1(Ref)		1(Ref)		1(Ref)	
T2	203/1885	0.55(0.44, 0.69)	<0.001	0.73(0.58, 0.91)	0.005	0.68(0.55, 0.85)	<0.001
T3	147/1870	0.48(0.37, 0.61)	<0.001	0.64(0.49, 0.82)	<0.001	0.55(0.43, 0.72)	<0.001
P for trend			<0.001		<0.001		<0.001
		CVD mortality					
Variable	No.death/total	Model1		Model2		Model3	
		HR (95%CI)	*P*-value	HR (95%CI)	*P*-value	HR (95%CI)	*P*-value
ALI, Per 10 U increment	211/5539	0.90(0.84, 0.95)	<0.001	0.93(0.89, 0.98)	0.004	0.89(0.84, 0.94)	<0.001
ALI tertiles							
T1	101/1784	1(Ref)		1(Ref)		1(Ref)	
T2	66 /1885	0.74(0.47, 1.15)	0.18	0.98(0.62, 1.54)	0.926	0.80(0.50, 1.26)	0.333
T3	44/1870	0.44(0.29, 0.66)	<0.001	0.56(0.36, 0.85)	0.007	0.41(0.26, 0.64)	0.001
P for trend			<0.001		0.017		<0.001

Abbreviations: ALI, advanced lung cancer inflammation index; Ref, reference; HR, hazard ratios; CI, confidence interval; CVD, cardiovascular disease; PIR, poverty income ratio; BMI, body mass index.

Model 1: unadjusted.

Model 2: age, sex, race, PIR, education level, physical activity.

Model 3: model2 + smoking status, BMI, CVD, diabetes, hypertension, inhaled corticosteroid.

Supplementary Table S4. Sensitivity analysis using E-values.

All -cause mortality		
Variable	Model 3<br>HR (95%CI)	*E*-vaule
ALI, Per 10 U increment	0.95(0.91, 0.99)	1.29
ALI tertiles		
T1	1(Ref)	
T2	0.68(0.55, 0.85)	2.30
T3	0.53(0.41, 0.68)	3.18
CVD mortality		
ALI, Per 10 U increment	0.90(0.85, 0.96)	1.46
ALI tertiles		
T1	1(Ref)	
T2	0.84(0.55, 1.28)	1.67
T3	0.47(0.31, 0.71)	3.68

Abbreviations: ALI, advanced lung cancer inflammation index; Ref, reference; HR, hazard ratios; CI, confidence interval; CVD, cardiovascular disease; PIR, poverty income ratio; BMI, body mass index.

Model3: age, sex, race, PIR, education level, physical activity, smoking status, BMI, CVD, diabetes, hypertension, inhaled corticosteroid.

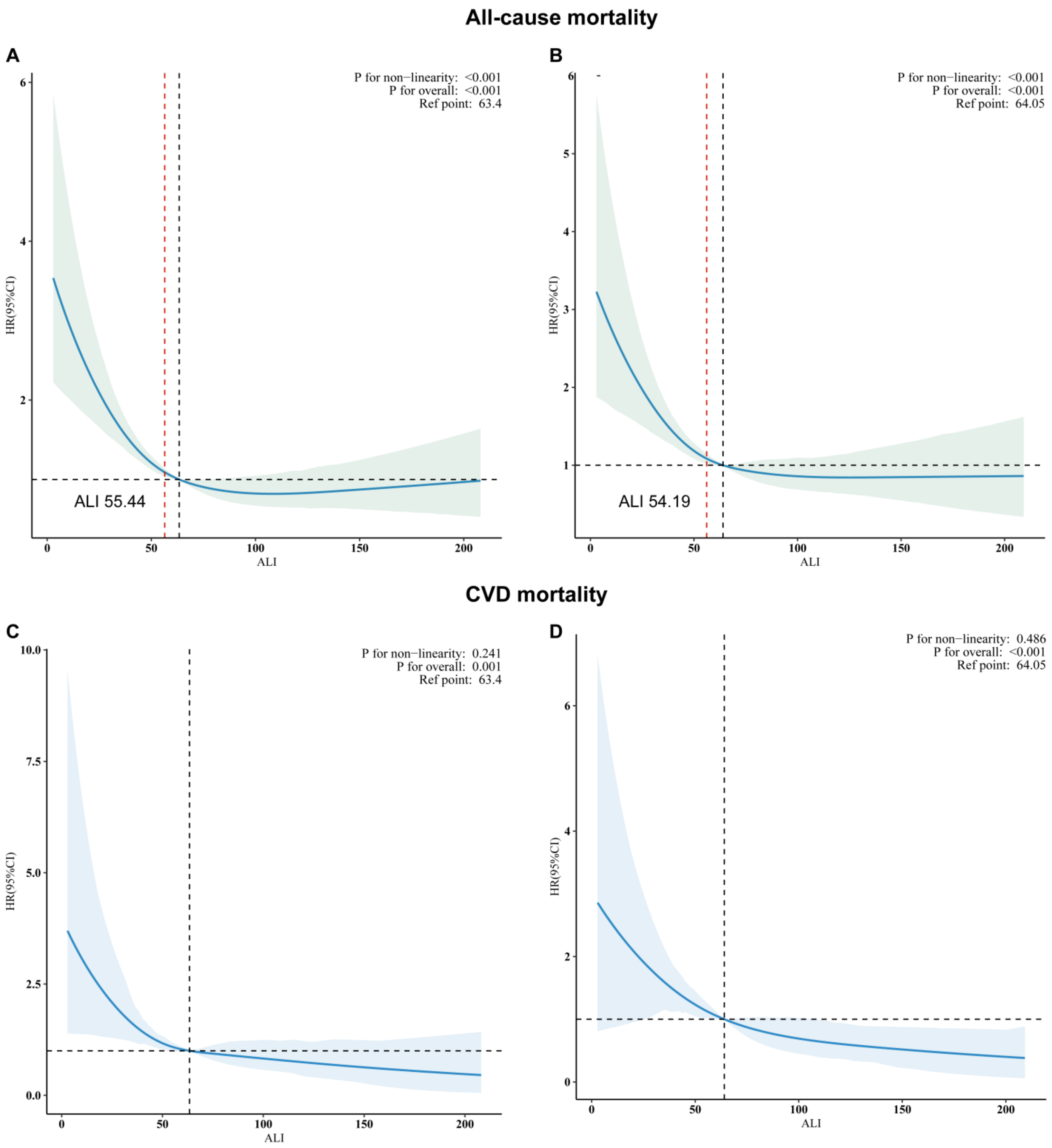

Supplementary Figure S1. Association between ALI and all-cause and CVD mortality in asthma patients. (A, C) excluding individuals with missing covariate data. (B, D) excluding individuals with cancer. Adjusted for age, sex, race, PIR, education level, physical activity, smoking status, BMI, CVD, diabetes, hypertension, inhaled corticosteroid. Notably, 99% of the data are displayed in the figure. The solid line depicts the estimated values, accompanied by shaded ribbons representing the 95% confidence intervals. The horizontal dotted line indicates an odds ratio of 1.0, serving as the reference line. Furthermore, the red vertical dotted line signifies the ALI threshold value of 55.44, 54.19, while the black vertical dotted line marks the reference point, set at an ALI value of 63.4, 64.05. Abbreviations: ALI, advanced lung cancer inflammation index; CVD, cardiovascular disease; PIR, poverty income ratio; BMI, body mass index.

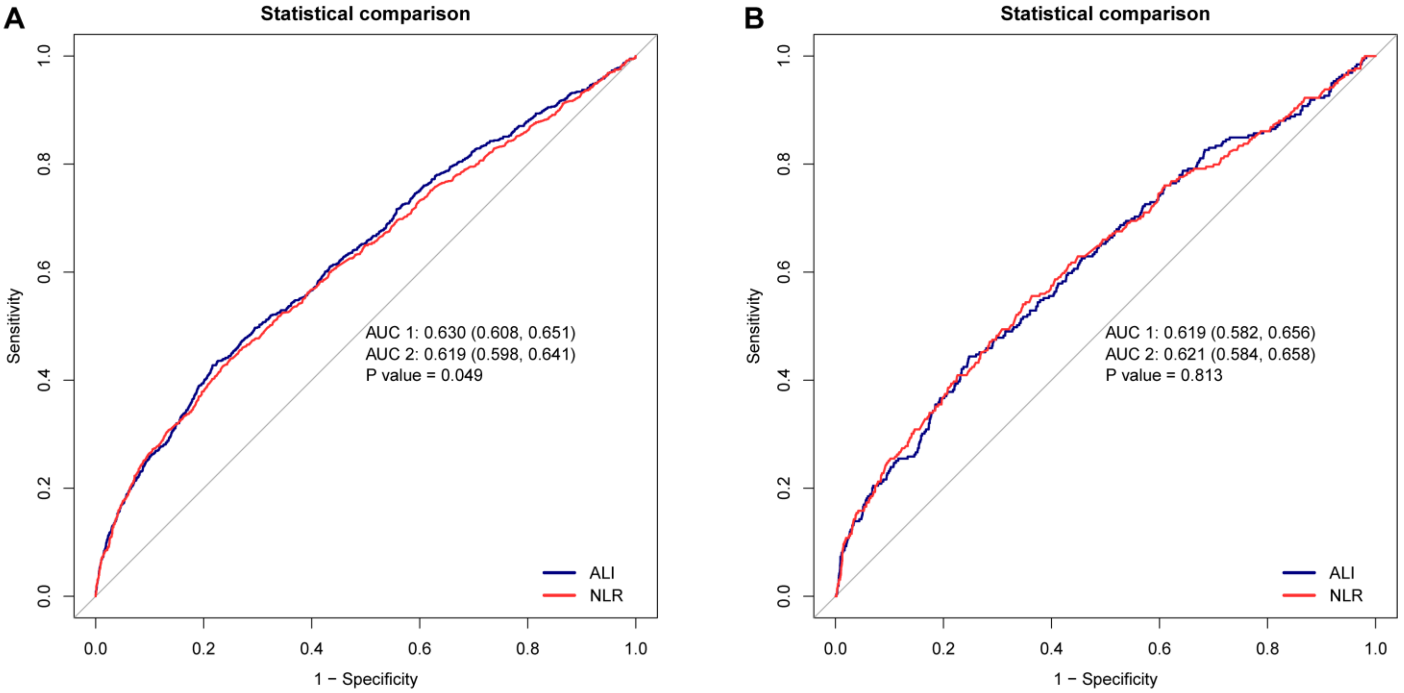

Supplementary Figure S2. Comparative ROC analysis of ALI and NLR for predicting mortality outcomes. Figures A and B illustrate the ROC curves for ALI and NLR in predicting all-cause (A) and Cardiovascular Disease (CVD) mortality (B). ALI, advanced lung cancer inflammation index; NLR, neutrophil-to-lymphocyte ratio.
